# Heterologous mesenchymal stem cells successfully treat femoral pseudarthrosis in rats

**DOI:** 10.1186/1479-5876-10-51

**Published:** 2012-03-20

**Authors:** Manoel Luiz Ferreira, Paulo Cesar Silva, Lucas Henrique Alvarez Silva, Danielle Cabral Bonfim, Lucas Cristo Conilho Macedo Müller, Christiano Costa Espósito, Alberto Schanaider

**Affiliations:** 1 Post-graduate Program in Surgical Sciences, Department of Surgery, School of Medicine, Federal University of Rio de Janeiro, Rio de Janeiro, Brazil; 2 Center of Experimental Surgery, Department of Surgery, School of Medicine, Federal University of Rio de Janeiro, Rio de Janeiro, Brazil; 3 Post-graduate Program in Morphological Science, Biomedical Science Institute, Federal University of Rio de Janeiro, Rio de Janeiro, Brazil; 4 FAPERJ-Fundação Carlos Chagas Filho de Amparo à Pesquisa do Rio de Janeiro, Rio de Janeiro, Brazil; 5 National Council for Scientific and Technological Development - CNPq, Brasília, Brazil; 6 Centro de Cirurgia Experimental-CCS, Avenida Carlos Chagas Filho 373, Bloco J, 2° andar, Cidade Universitária, Ilha do Fundão, Rio de Janeiro 21941-902, Brazil

**Keywords:** Rats, Pseudarthrosis, Osteotomies, Animal disease models, Bone marrow, Stromal cells

## Abstract

**Background:**

This study evaluated the effectiveness of treating pseudarthrosis in rats by using bone marrow cell suspensions or cultures of bone marrow mesenchymal stromal cells

**Methods:**

Thirty-eight specific pathogen-free (SPF) animals were randomly assigned to four groups: Group 1, Control, without surgical intervention; Group 2 (Placebo), experimental model of femoral pseudarthrosis treated only with saline solution; Group 3, experimental model of femoral pseudarthrosis treated with heterologous bone marrow cells suspension; Group 4, experimental model of femoral pseudarthrosis treated with cultures of heterologous mesenchymal stromal cells from bone marrow. When pseudarthrosis was confirmed by simple radiological studies, digital radiography and histopathology after a 120-day postoperative period, Groups 2, 3 and 4 were treated as above. At 30, 60 and 90 days after the treatment, all animals were evaluated by simple radiological studies, and at the end of the experiment, the animals were assessed by computed axial tomography and anatomopathological and histomorphometric examinations.

**Results:**

Injected cells were detected in the areas affected by pseudarthrosis using scintigraphy within the first 24 hours after their administration. After 60 days, the animals of Group 3 showed callus formation while the animals of Group 4 presented periosteal reaction and had some consolidated areas. In contrast, Group 2 showed a predominance of fibro-osteoid tissue. After 90 days, bone consolidation and remodeling was observed in all animals from Group 3 whereas animals from Group 4 exhibited partial consolidation and those ones from Group 2 persisted with pseudarthrosis.

**Conclusion:**

The treatment with heterologous bone marrow cells suspension proved to be effective in the treatment of pseudarthrosis whereas cultures of heterologous bone marrow mesenchymal stromal cells did not show the same potential to aid bone healing.

## Background

The term pseudarthrosis defines the nonunion of a fracture when the bone repair process has ceased for any reason. The lack of union of fractured bone surfaces in such circumstances results from the presence of fibrocartilage or fibrous tissues between the bone extremities [1]. Other factors that contribute to the persistence of this problem are the instability of the fracture, the lack of adequate blood supply and the presence of infection.

The fracture healing process usually begins from a trauma that results in hematoma formation, followed by inflammation, angiogenesis, cartilage formation, calcification, and bone remodeling with the absorption of the newly formed callus. The bone consolidation process requires growth factors, mesenchymal stem cells (MSCs) and cytokines [2].

To date, there is no optimal technique for the treatment of pseudarthrosis; therefore, surgery is still the best option [3]. However, cell therapy has become a promising alternative because it is less invasive than surgery. Thus, stem cells (SCs) have been tested in bone diseases because of their capacity to self-renew and generate differentiated cells to repair specific tissues [4-6]. Among the various types of bone marrow cells, adult mesenchymal stem cells (MSCs) have sparked interest. Their multipotentiality and the techniques available for their isolation and in vitro expansion have validated the use of these cells as a therapeutic alternative with a broad spectrum of applications in cases of bone fractures and chronic bone lesions [6,7].

The current study evaluated the efficacy of heterologous bone marrow cells suspension and cultures of heterologous bone marrow mesenchymal stromal cells in the treatment of hypertrophic pseudarthrosis considering that both types of cells have the ability to generate a new bone after in vivo transplantation. Cells suspension has a heterogeneous population which contains all lineages from the bone marrow including progenitor and hematopoietic stem cells and also bone marrow mesenchymal stromal cells. On the other hand cultures of bone marrow stromal have cells with a stem-cell-like character. It is still controversial whether bone marrow stromal cells and so-called mesenchymal stem cells are the same population [8].

## Methods

This study was approved by the Ethics Committee for the Use of Laboratory Animals in Research of the School of Medicine of UFRJ, in accordance with the Brazilian legislation and the NIH Guide for the Care and Use of Laboratory Animals.

### Animals

A total of 38 specific pathogen-free (SPF) male *Rattus norvegicus albinus*, aged between four and six months with an average weight of 250 g, were obtained from the vivarium of the Experimental Surgery Center at the School of Medicine of UFRJ. The animals were maintained at 25°C and under a photoperiod of 12 hours (light/dark cycle). They were provided water and pellet of balanced industrial food ad libitum.

Among the 38 experimental animals, 6 served as donor of bone marrow cells. The other 32 animals were randomly divided into four groups, each containing eight animals: Group 1 (Control), without operative procedures, only observed during the experiment. Group 2 (Placebo), in which pseudarthrosis was treated with saline solution; Group 3, in which pseudarthrosis was treated with heterologous bone marrow cell suspension; and Group 4, in which pseudarthrosis was treated with cultures of heterologous bone marrow mesenchymal stromal cells.

The same anesthetic mixture (10% ketamine,10 mg/kg and 2% xylazine, 100 mg/kg) was administered intraperitoneally before the two surgeries conducted for the production of pseudarthrosis and the injection of saline solution (Group 2) or bone marrow cells (Groups 3 and 4).

### First surgical intervention

**Figure 1:**
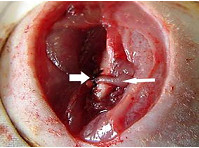
**Fascia lata (narrow arrow) interpositioned between the aligned bone segments and tied with nylon 3-0 wire (large arrow)**.

A model of pseudarthrosis previously designed by one of the authors of this study was used [9]. This model consisted of the following surgical steps: diaphyseal osteotomy of the left femur, interposition of a pedicled fascia lata flap sutured to the distal portion of the semimembranosus muscle, transfixion of a 3-0 nylon wire in the proximal and distal segments, bone alignment of the two extremities with the aid of a knot tightened onto the nylon wire and layered closures as the final step (Figure 1).

After the 120-day postoperative period, in addition to performing simple radiological studies (using the Heliodent 60B device, Siemens, 127 V-60 kV, 10 mA, 50-60 Hz, for 2 seconds), three animals from each group were painlessly euthanized with an anesthetic overdose (4 ml of 2.5% sodium pentothal delivered. intraperitoneally). The area affected by pseudarthrosis was then removed for analysis with a digital X-ray device (GE Silhouette^® ^model, 20 kV, 0.41 mA, for 180 seconds), followed by a histopathological study with hematoxylin and eosin stain.

### Degree criterion of claudication assessment

The authors adapted some criterion of claudication, generally used for dogs (Table 1), and classified them according to 5 degrees of severity (from absence to severe) [10].

**Table 1:** Classifications of claudication

Degree	Criterion
1	Absence of claudication, full limb support when the animal was in a standing position or during physical activities.

2	Mild claudication after exercise or prolonged decumbency.

3	Sporadic claudication when walking or running and weight relief on the operated limb, even in a standing position.

4	Constant claudication when walking and lack of limb support when running; incomplete support in the orthostatic position.

5	Full or absent support during physical activities or in a standing position.

Source: Adapted from Muzzi (2003)

### Collection of cell suspensions and cultures of bone marrow cells

Cells were collected from the femur and tibia of six donor animals after underwent CO_2_-induced euthanasia. The bones were then manually cleaned of muscle tissue and treated for 30 minutes at 37°C with a 0.125% trypsin solution and 1 mg/mL of collagenase IA (Sigma Aldrich Co., St. Louis, MO, USA) to remove fibrous tissue. Bone marrow collection was performed by sectioning the epiphyseal regions and flushing the internal cavities of the bones with culture medium using disposable syringes. The obtained cell suspension was filtered through a nylon mesh with a porosity of 100 μm and centrifuged at 1,500 rpm for 5 minutes. The cells were resuspended in low glucose DMEM (Dulbecco's Modified Essential Medium, Hyclone) and quantified in a hemocytometer. The cell concentration was adjusted, and 3 × 10^6 ^cells in 500 μL of DMEM, without fetal bovine serum (FBS), were injected into animals with 1-mL syringes. The remaining bone marrow cells, obtained according to the above described procedure, were distributed at a concentration of 1 × 10^6^/mL in culture flasks with 25 cm3 of low glucose DMEM containing 15% FBS (Cultilab, Campinas, SP, Brazil) and antibiotics (100 U/ml penicillin G sodium and 100 μg/mL streptomycin, Sigma Aldrich Co., St. Louis, MO, USA) and incubated at 37°C with 5% CO_2 _for two or three days. After this period, any nonadherent cells were removed by washing the monolayer with phosphate-buffered saline solution (PBS), and the culture medium was replaced. Cells were maintained in culture until they reached 70% confluence and expanded up to the third passage. Cells were cryopreserved in liquid nitrogen at -140°C until they were needed; at this time, they were thawed in a 37°C water bath and centrifuged at 1,500 rpm for 5 minutes. Cell viability was determined using the Trypan Blue Exclusion Test. Each animal received injections of 3 × 10^6 ^cells in 500 μL of DMEM.

### Second surgical intervention

To access the areas affected by pseudarthrosis in Groups 2, 3 and 4, a 5-cm long longitudinal incisions were made in the diaphysis of the femur, which was dissected by layer under strict hemostasis. The fibrous tissues found on and around the fractured area were excised, and the medullary canal of both extremities of the femur was made permeable by making rotational movements with a hypodermic needle (27 mm × 8 G). Animals in Groups 3 and 4 received injections of cells suspension and cultures of mesenchymal stromal cell, respectively, that were extracted from bone marrow and marked with technetium-99 m radionucleotide (using the protocol of the Laboratory of Cell and Molecular Marking of the Department of Radiology and Nuclear Medicine of Hospital Universitário Clementino Fraga Filho - UFRJ) [11]. These injections occurred in and around the medullary canals of fracture segments. The same procedure was conducted in Group 2, but with the administration of saline solution into the medullary canal.

The fracture was stabilized with the introduction of a flat intramedullary pin that occupied 50 to 60% of the diameter of the medullary canal. The surgery was concluded with muscle and skin closures.

At 30, 60 and 90 days after the second intervention, the degree of claudication was assessed, and the animals were classified according to the increasing scale of gravity [9] (Table 1).

At the same time points, all animals of Groups 3 and 4 (bone marrow cells treatments) and Group 2 (placebo) were submitted to an X-ray examination. All animals also underwent a CT scan (NewTom 3 G, QR, Italy, 100 μSv) 90 days after the second surgery. The images were captured with NNT software and analyzed with Dental Slice software to observe the line of calcification throughout each sagittal incision.

Samples of the left femur and the tissues surrounding the affected areas of pseudarthrosis were collected for histopathological analysis. The material was fixed in 10% buffered formalin, decalcified with Allkimia^® ^and routinely processed for paraffin embedding. The histological samples obtained were stained with hematoxylin and eosin (HE) stain and evaluated using the 10× objective of a Leica^® ^DM5000 B binocular optical microscope. A digital camera attached to the microscope was used to sequentially capture images (10 per slide). Photomicrographs were analyzed using Image Pro Plus ^® ^4.5 software (Media Cybernetics) to quantify at histomorphometry the percentages fibrous tissue of each sample.

### Statistical analysis

For the claudication results, the Mann-Whitney U-test was applied. To analyze the histomorphometric data, the nonparametric Kruskal Wallis test was applied, followed by the Dunn's test, with the aid of the GraphPad InStat^® ^(v. 3.01) software. The results were considered significant for all p-values under 0.05.

## Results

In all animals, except Group 1, claudication reached its maximum level (with an average score of 5) after the first surgery, and this maximum level was maintained for 30 days after the second surgery. Ninety days after the injection of bone marrow cells, a slight amount of claudication was observed in Group 3 (grade 2), whereas in Group 4, it was worse with a constant level of claudication including both the absence of limb support to run and incomplete limb support in the standing position (grade 3). Group 2 maintained a high degree (grade 4) of claudication. Results in Group 3 were significantly different than Groups 2 and 4 (p < 0.05).

**Figure 2:**
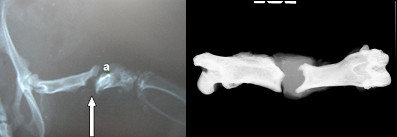
**Plain radiography (A) and digital radiography (B) of the femur of a rat specimen**. The white arrows indicate the formation of pseudarthrosis 120 days after the first surgery. This result was found for all animals in Groups 2, 3 and 4.

**Figure 3:**
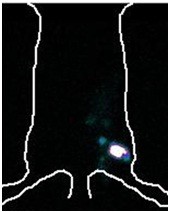
**Scintigraphic image captured at 24 hours (Groups 3 and 4) after the stem cells treatment and the outline of a rat**. Note the whitish area with a red halo that delineates the concentration of bone marrow mesenchymal cells in this area.

Plain and digital radiographies performed 120 days after the first surgery revealed pseudarthrosis formation as a result of the model used in rats of Groups 2, 3 and 4 (Figure 2). In Groups 3 and 4, twenty-four hours after treatment, scintigraphies showed an uptake of bone marrow mononuclear cells in the area of pseudarthrosis (Figure 3).

**Figure 4:**
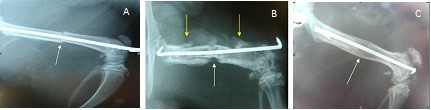
**Plain film of the femur 90 days after the injection of saline solution (Group 2, placebo) or stem cells (Groups 3 and 4)**. The absence of bone consolidation in Group 2 rats (A) was verified. Group 4 animals (B) had periosteal reactions (yellow arrows) but did not have bone calluses (white arrow). In Group 3 (C), bone remodeling with callus absorption (arrow) was observed.

In the plain radiological study of the femurs from animals in Groups 2 and 4 performed 30 days after the second surgery, no calcification was detected between the fractured segments. At the same time point, animals from Group 3 showed early calcification. After 60 days, callus formation was detected in Group 3, whereas in Group 4, there was an intense periosteal reaction with some consolidated areas. At 90 days after surgery, Group 3 animals exhibited bone remodeling and callus absorption, whereas the Group 4 results did not differ from the results observed at 60 days. In Group 2 animals, no bone consolidation was observed throughout the study (Figure 4).

**Figure 5:**
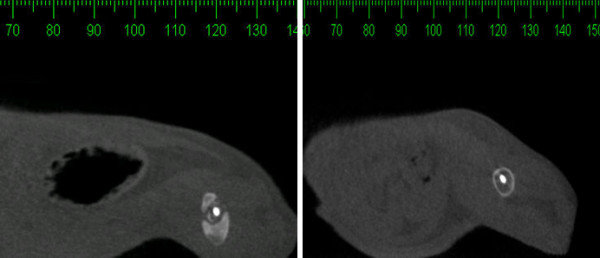
**Tomographic image of a sagittal section of a femur 90 days after the second surgery**. A pseudo-halo formation was detected in Group 4 animals (A), the consolidation of which was partial; in turn, Group 3 rats (B) had full-halo consolidation around the intramedullary pin, which represents bone consolidation.

For Group 2, the computed tomography images made on the sagittal incisions, 90 days after the second surgery, showed an absence of bone halo, unlike Group 3, in which there was complete bone consolidation represented by a halo around the entire bone surface in the previously fractured region (Figure 5). In Group 4 specimens, the halo was incomplete.

**Figure 6:**
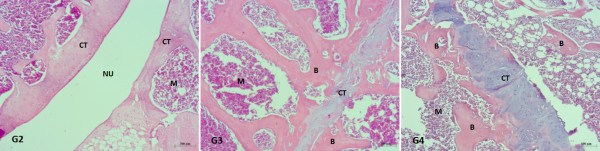
**Histological preparations 90 days after the second surgery, HE, using a 10× objective and the scale bar is 100 μm, in groups 2, 3 and 4**. G = Groups, NU = nonunion area, CT = cartilage, B = bone. M = medulla. There was no bone consolidation in Group 2. In Group 3, almost all of the fractured area was filled with bone, with a small area of cartilage interposed. In Group 4, there was partial bone consolidation, with a significant amount of cartilage in the fracture line.

**Figure 7:**
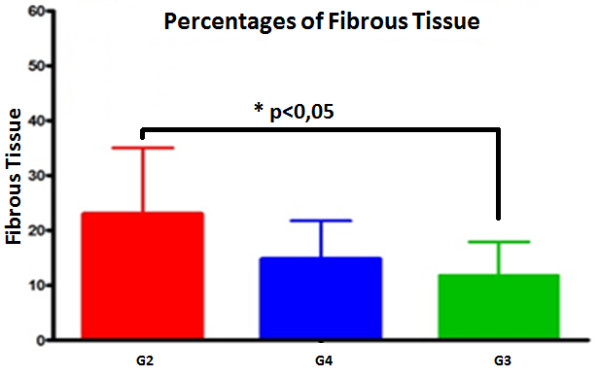
**The percentages of fibrous tissue formed 90 days after the injection of stem cells or placebo**. There was less fibrosis in the group (G) that received a suspension of bone marrow cells (G3) as compared with Group 2 (G2, placebo). * G2 vs. G3 = p < 0.05.

The histopathological results obtained 120 days after the first surgery demonstrated the absence of bone consolidation in all animals. Ninety days after the second surgery, an absence of bone was observed in Group 2, bone remodeling was observed in Group 3 and partial bone consolidation interspersed with a significant amount of cartilage was detected in Group 4 (Figure 6). The histomorphometric analyses revealed an increase in the fibrotic area in Group 2 as compared with Group 3 90 days after the second surgery (Figure 7).

## Discussion

Traumas that result in bone fractures trigger a repair process that is highly associated with osteoblast proliferation and substances inhibit the fracture-induced inflammatory process. As a result of this inflammation, there is active mitochondrial oxygen consumption and the production of reactive oxygen species [12]. Under these circumstances, viable osteoprogenitor cells must be present to allow for the production of an extracellular matrix at each stage of bone repair [13].

The mechanism of bone remodeling is not clear, and there is lack of consensus in the literature. The osteogenic lineage, which includes both osteoblasts and chondroblasts, is derived from a stromal stem cell in the postnatal organism [14]. However, studies indicate that agents that induce osteoclasts are necessary to allow this process to occur in the proper manner [15].

Several methods have been used to treat pseudarthrosis: decortication or osteoperiosteal scaling, cancellous autografting, the addition of bone morphogenetic protein, platelet growth factors, callus distraction with external fixators, vascularized bone graft filling, internal stabilization materials (a compression plate, pin or locked reamed intramedullary nailing), external stabilizers (orthotics or external fixators), ultrasound, shock waves and electromagnetic fields [16-20]. This plethora of options indicates a lack of consensus on the best method to treat pseudarthrosis and results in unfitness for work and deformities in those patients who experience pseudarthrosis, causing heavy psychosocial and economic burdens for families and overcrowding in public health stations.

Within the scope of experimental studies, it is very difficult to reproduce pseudarthrosis bone structures in laboratory animals because the models are generally accompanied by significant injuries in surrounding tissues and in the bone itself [21,22], creating biases in the interpretation of results. SPF Wistar rats were chosen because of their small size and easy handling, which also applies to the use of intramuscular anesthesia; the existence of a bony structure covered with a large muscle mass in the hind limbs, which is comparable to that of human species; and their reduced susceptibility to infection.

A reliable model of hypertrophic pseudarthrosis in animals that was previously developed by the authors was able to mimic the conditions found in human beings [9,23]. Pseudarthrosis was detected in all animals within an acceptable period, without the presence of infectious foci. The simple bone section without fracture reduction is not itself sufficient to prevent spontaneous recovery because there may be calcification even in the presence of a misalignment in the bone axis [24]. Given this possibility, the interpositioning of a pedicled fasciae lata muscle flap was an essential step to ensure success in obtaining nonunion bone in all cases. Regarding this matter, no similar descriptions for such surgical technique were found in the literature.

There are other models designed for small and medium-sized animals that reproduce pseudarthrosis, but these models involve the immediate treatment of fractures with the placement of fasteners; also, some are difficult to manufacture, expensive and require the eventual removal of bone segments of different lengths to artificially create a gap [24-29]. Sometimes such models also holds for the cauterization or resectioning of the periosteum [29]. The application of these models to humans is limited because the mechanism of injury often creates conditions that are different from these that occur after a trauma. Furthermore, the areas of fracture are immediately immobilized without generating instability and without the interpositioning of fibrocartilaginous tissues in the initial phase of the healing process.

Indeed, in all animals of Groups 2, 3 and 4, the clinical evaluations showed severe claudication and also the histopathological examinations detected the presence of fibro-osteoid tissue 120 days after the first surgery, indicating pseudarthrosis. As observed by other authors, the most significant findings were the absence of endochondral ossification, the prolapse of soft tissue parts and the closure of the medullary canal [26-28]. These characteristics were also detected in plain and digital radiological studies, with the presence of hypertrophic pseudarthrosis in all of the animals subjected to femoral fractures.

Cell therapy has generated a great interest in the design of animal models intended to investigate new options for the treatment of pseudarthrosis. Recently, studies have shown that multipotent stem cells can differentiate into lineages other than those of the tissue of origin [30-32]. As of yet, the risks of treatment with stem cells are poorly understood (e.g., disease transmission, neoplasia formation, immune responses with rejection, nidation in unexpected in places, atypical features); therefore, *a priori *studies with animal models are essential [33].

Mesenchymal stem cells (MSCs) are a population of adult cells firstly described in the bone marrow. From a physiological point of view, the differentiation of MSCs into osteoblasts is fundamental for the organization and repair of bone tissues [31]. Most clinical trials involving stem cell therapy have used mesenchymal cells derived from bone marrow [33].

The presence of cellular elements of bone marrow, including vascular growth factors, is important in promoting angiogenesis and essential for bone regeneration [34,35]. Tissue revascularization facilitates the transformation of pluripotent cells into osteoblasts and those cells that cross the fracture lines enable primary Haversian remodeling [5,18,36]. For this reason, care was taken not only to revive the edges of the wound by removing fibrotic tissues in and around the area of pseudarthrosis but also to recanalize the medullary region, leaving about 50 to 60% of the diameter of this canal free.

The recovery of functional osteoblasts from MSCs is a widely used technique in tissue engineering and cell therapy. A study conducted by Iida and colleagues involving more than 300 rats and the use of *in vitro *autologous mesenchymal cultures for bone reconstruction after surgical excision revealed that MSCs have an osteogenic potential [37].

The vast majority of studies involving mesenchymal stem cells are performed *in vitro*. Although the biology of these cells in culture is fairly well known, the same is not true for fractured bones under *in vivo *conditions [38,39].

MSCs can be grown in *in vitro *cultures and differentiated into osteocytes, chondrocytes or other cells [40]. To achieve good quality regenerative processes, at least 2 × 10^6 ^cells must be transplanted, which can be accomplished without the need for culturing MSCs [40,41]. However, other studies on bone regeneration argue that osteogenesis can be benefited by techniques that concentrate osteoprogenitor cells [42]. Osteoprogenitor cells derived from the mesoderm and mesenchymal cells have a mitotic capacity and produce growth factors. The use of cultures enables replication with long-term cell maintenance. In Group 3 it was injected bone marrow cells suspension containing a pool of hematopoietic and non-hematopoietic lineage including stem cells and mesenchymal stromal cells. Adult stem cells are rare and nearly 1 in 10,000 to 15,000 cells in the bone marrow is a hematopoietic stem cell [43]. In Group 4 bone marrow mesenchymal stromal cells was implanted but it is not clear if such stromal cells could be classified as stem cells or progenitor cells. In fact to characterize the cellular lineage in vitro, a population of pure stem cells from bone marrow should be isolated and a reproductive cloning and expansion should be done in culture dishes to induce the differentiation of bone marrow stromal cells to osteoblastic lineage. However it has been described at clonal level, a progressive loss of stem features with increasing population doubling in culture [7].

The associations among mesenchymal cells, bone morphogenetic proteins and growth factors seem to favor their use in the treatment of hard-healing bones [30]. Seeking to prevent bias in the interpretation of results, the use of osteoinductive substances such as bone morphogenetic proteins was avoided because our focus was the therapeutic potential of mesenchymal stem cells.

Bone marrow cells suspension are considered the best choice because, compared with cultures of bone marrow mesenchymal stromal cells, that procedure is more cost effective, can be more easily accomplished and requires less time and less technical instrumentation [41]. Despite the use of heterologous cells in this experiment, there was no need for any prior immunosuppressive therapy. No sign of severe acute or chronic inflammation was observed in animals injected with bone marrow cell suspensions. Besides, no mononuclear infiltration that is characteristic of chronic immune-mediated inflammation was observed in histopathological sections.

In general, the systemic vascular compartment is targeted in the administration of stem cells for therapeutic purposes [30]. These cells have chemotactic migratory abilities to travel to injured areas; in addition, these cells settle in the impaired areas. This feature is known as homing [12]. Nevertheless, many of these cells are initially retained in the lung and the liver [30]. Thus, we administered the bone marrow cells directly into the lesion after the surgical release of fibrosis and medullary recanalization. The location of such cells was confirmed by scintigraphic examinations.

In Group 3, there was bone consolidation within 60 days after the administration of bone marrow cells suspension. This result was also partially observed within 90 days in Group 4 animals, which were treated with cultures of stromal cells, and there was an intense periosteal reaction. Clinical examinations, imaging studies and histomorphometric analyses confirmed these findings endorsing the best efficacy of the cells in suspension.

In a fracture pseudarthrosis model involving rats, Nakamura and colleagues conducted a therapeutic study of the effect of the transplantation of a single layer of bone marrow cells cultured in a medium containing dexamethasone and ascorbic acid phosphate. Radiographs and histological analyses were performed at 2, 4 and 8 weeks, while ultrasound and biomechanical analyses were performed eight weeks postoperatively. In the group with stromal cells, radiographs and histological sections demonstrated the formation of callus areas around the fracture site. In contrast, the control group, which did not receive the transplantation, exhibited pseudarthrosis of the femur. These authors concluded that cell transplantation in a single layer may contribute to the reconstruction of bone tissues in cases of pseudarthrosis, bone defects and osteonecrosis [32]. Our study examined a greater interval, 12 weeks after the administration of bone marrow cells suspension stimulated complete bone healing in rats with hypertrophic pseudarthrosis, whereas cultures of bone marrow mesenchymal stromal cells were less effective. The biomechanical analyses of animals in Group 3 indicated a significant and early recovery with regard to the walking capacity of rats.

Considering the high socioeconomic costs resulting from the inability to work, the development of disabilities that result from pseudarthrosis, and the lack of consensus regarding the best treatment of pseudarthrosis, bone marrow cells suspension therapy seems to be a promising option. Further studies that examine the administration of these cells by less invasive means or even with a specific lineage of stem cells expanded in cultures are needed for the purpose of delineating their therapeutic applicability in pseudarthrosis in human beings to achieve more favorable treatment outcomes.

## Conclusions

Bone marrow cells suspension appear to be more effective in the treatment of pseudarthrosis than cultures of bone marrow mesenchymal stromal cells because they seemingly contribute to the successful bone remodeling and reduce recovery times.

## Competing interests

The authors declare that they have no competing interests.

## Authors' contributions

MLF made substantial contributions to the acquisition, analysis and interpretation of acquired data. He also developed the experimental model and was involved in drafting the manuscript. PCS participated in the in vivo experimental model and made substantial contributions to the design of the study. LHAS helped with the elaboration of the animal experimental model. DCB helped with the preparation and acquisition of the stem cells and also wrote the protocol for the preparation of the stem cells. MIDR contributed to the design and scientific background of cell-based therapy protocol, helped with cell processing and culture and with the acquisition of histopathological data. LCCMM helped with the elaboration of the animal experimental model. CCE took care of the vivarium and controlled the entire experimental environment for the duration of the experiment. AS made substantial contributions to the conception and design of data acquisitions and analyses and was involved in critically revising the manuscript as well as providing the final approval of the version to be published. All authors read and approved the final manuscript.

## Authors' information

MLF: Post-graduate student in Surgical Sciences, Department of Surgery, School of Medicine, Federal University of Rio de Janeiro (UFRJ) and Assistant Professor at State University of Santa Cruz, Bahia, Brazil

PCS: Associate Professor and Veterinarian at the Center of Experimental Surgery, Surgical Department, School of Medicine of UFRJ, Brazil

LHAS and LCCMM: Undergraduate student, School of Medicine, UFRJ, Brazil

DCB: Post-graduate student, Laboratory of Cellular Biology, UFRJ, Brazil

CCE: Veterinarian and scholar at Carlos Chagas Filho Research Support Foundation - FAPERJ, Rio de Janeiro, Brazil

AS: Full Professor and Coordinator at the Center of Experimental Surgery and the Post-graduate Program in Surgical Sciences, Department of Surgery, School of Medicine, UFRJ. Researcher of FAPERJ and the National Council for Scientific and Technological Development) - CNPq, Brazil
